# Newspaper Recipes

**Published:** 1870-04

**Authors:** 


### NEWSPAPER RECIPES.

We are frequently called upon to notice ill results from the administration of medicines, that have been recommended, for positive cures, in the columns of our newspapers. Editors are constantly in the habit of transferring to their columns any little recipe for the cure of disease, that they may find going the rounds of the press, without ever stopping to enquire into its merits; and so, often, giving publicity to so-called means of cure that had much better been suppressed.

Recently a lady read, in one of the public prints, that turpentine dropped in the ear, would instantly cure ear-ache. She thoughtlessly tried the remedy on her little girl, and not only destroyed her hearing, but rendered her so ill from inflammation of the internal ear, that her life was despaired of for a long while. We have frequently seen difficulties of hearing produced, by placing red-pepper, done up in cotton, into the ear for the cure of ear-ache—a popular remedy, which we have noticed published in a score of country newspapers.

We have before us a quantity of these newspaper formulæ. Some of them recommend "Fowler's Solution" for rattle-snake bites—a remedy that is as dangerous in unskillful hands as the poison of the snake. Another advises belladonna, in opium poisoning,—one poison being as fatal as the other, unless administered by the physician. Indeed, we read daily, in our secular exchanges, all manner of absurd and often absolutely *dangerous* advice upon medical and surgical subjects; and, if one-half of them are carried out by those who read them, we have no reason to wonder at our increased mortality reports.

Recently we read an article in that usually reliable journal, Harper's *Bazar*, upon foreign substances in children's ears. If the directions there given, for displacing such bodies, were strictly complied with, we will guarantee the destruction of hearing in nine-tenths of the ears so manipulated.

The article reads:—"Having detected its position and its nature, it, if soft, will be most easily removed by an *instrument sharp enough to pierce it;* if hard, by the ordinary *spoon attached to the tweezers found in most toilette-cases.*" Now, we don't know who the author is of the articles headed "Before the Doctor Comes," in Harper's *Bazar*, but we do know that he has no business to offer *such* advice for the removal of foreign bodies from the ear. Any physician who has ever attempted the removal of these substances, can testify that such a procedure would be exceedingly hazardous; for, the attempt to place a sharp instrument into the offending body, or still, to introduce a "spoon" behind it, would only tend to thrust it further into the ear; and a persistent trial to so remove it, would bring the substance in contact with the delicate *drum* of the ear, either severely injuring or perhaps rupturing it. The advice given further on in the article referred to, we like better—it is good common sense—viz: "If these means fail, take a common syringe, and first inject into the passage a little sweet oil, and, subsequently, some water, (warm) with sufficient force to drive it behind the object, which will probably be floated out on the reflux." Children are continually in the habit of thrusting peas, beans, kernals of corn, buttons, pebbles and other substances into their ears and noses. If these articles cannot be removed by a careful system of syringing, with warm water, no probing should be done with a view to their removal. It is altogether too dangerous a proceeding, and when necessary, should only be done by the physician, the advice of the contributor to the *Bazar*, to the contrary, notwithstanding.

Finally, we desire to warn our readers against the use of remedies and nostrums which they find recommended in the newspapers. All editors are not physicians, by any means—therefore are not competent to give advice upon medical subjects. Remember that a puff for a patent medicine is *always* paid for, and that it is not reasonable to suppose that editors have taken all the medicines that they recommend to their readers, else they would not be so lively and sprightly as we are in the habit of seeing them. Domestic formulæ are apt to become *mixed* in their various copyings from one journal to another, therefore, are unreliable. Be careful what sort of medicine you take, and by whom given—it is dangerous stuff when not properly administered.

